# Dynamic models of occupational fatigue: a systematic review for risk management advancement

**DOI:** 10.1186/s12889-026-28156-9

**Published:** 2026-06-29

**Authors:** A. Wahyuni, Syamsiar S. Russeng, Agus Bintara Birawida, Hasnawati Amqam, Muhammad Arsyad Rahman, Ilham Bakri

**Affiliations:** 1 Department of Occupational Health and Safety, Faculty of Public Health, Hasanuddin University, Makassar, Indonesia; 2 Department of Environmental Health, Faculty of Public Health, Hasanuddin University, Makassar, Indonesia; 3 Department of Health Promotion, Faculty of Public Health, Hasanuddin University, Makassar, Indonesia; 4 Department of Industrial Engineering, Faculty of Engineering, Hasanuddin University, Makassar, Indonesia

**Keywords:** Occupational fatigue, Dynamic modelling, Fatigue risk management

## Abstract

**Background:**

Occupational fatigue is a major safety and health risk across work sectors. Because fatigue accumulates and fluctuates over time, effective fatigue management requires dynamic approaches that can anticipate risk and inform preventive controls rather than rely on point-in-time assessment. Although dynamic fatigue modelling has expanded rapidly, evidence remains fragmented across modelling paradigms and sectors, limiting clarity on which approaches are most suitable for implementation. This systematic review synthesises recent evidence on dynamic models of occupational fatigue and evaluates their relevance for fatigue management and risk control.

**Methods:**

Records published from 2019 to 2025 were identified from Scopus using DOI-indexed retrieval and screened with Watase Uake Tools to support article mining and Preferred Reporting Items for Systematic Reviews. Searches were complemented in PubMed, ProQuest, Wiley Online Library, and ScienceDirect. References were managed in Zotero, and screening, selection, and data extraction followed a structured process with manual searching and citation tracking.

**Results:**

From 180 records, 12 peer-reviewed studies met the inclusion criteria. Four modelling approaches were identified: physiology-informed dynamic simulation, machine learning and deep learning for near real-time monitoring, probabilistic dynamic models such as Dynamic Bayesian Networks, and symptom-network or Bayesian graph approaches. Models were most useful for fatigue management when they integrated multimodal inputs and produced actionable outputs such as fatigue risk levels and intervention triggers, but implementation was limited by small samples and insufficient field validation.

**Conclusion:**

Future research should prioritize longitudinal workplace validation, harmonized benchmarking, and deployable hybrid models integrating physiological, psychosocial, and contextual determinants.

## Introduction

Occupational fatigue is increasingly recognized as a major occupational safety and health hazard across sectors, especially in safety-sensitive and high-demand work systems. Evidence in the last five years links long working hours and insufficient recovery with adverse health outcomes and elevated risk, reinforcing fatigue as a priority for prevention and control in workplaces [1–3]. The World Health Organization and the International Labour Organization reported that long working hours are associated with increased mortality from cardiovascular outcomes, underscoring fatigue-related exposures as a population-level concern [1, 2].

A key challenge for prevention is that fatigue changes over time rather than remaining constant. Work schedules with night duty, extended shifts, overtime, and limited rest create conditions where fatigue can accumulate across hours and days and fluctuate within a single shift [3]. Recent occupational research frames work-related fatigue as a meaningful hazard for working populations, particularly when recovery opportunities are constrained [3]. Evidence from working-time research also shows that long and late working patterns combined with insufficient rest are associated with higher probability of occupational accidents, highlighting the operational consequences of time-dependent fatigue risk [4].

In response to the need for systematic control, fatigue risk management has increasingly been framed within structured safety management approaches. A rapid review by Safe Work Australia emphasizes that fatigue risk management is evolving alongside advances in sleep and circadian science and highlights the complexity of managing fatigue exposures arising from work factors, sleep opportunity, and recovery [5]. In safety-critical domains such as aviation, contemporary guidance defines a Fatigue Risk Management System as a data-driven means of continuously monitoring and managing fatigue-related safety risks using scientific principles and operational experience, with the aim of ensuring adequate alertness [6, 7]. While FMRS principles are well established, their effective implementation depends on robust analytical tools capable of translating fatigue-related data into predictive and actionable insights. Therefore, this review focuses on evaluating dynamic modelling approaches that can support FRMS by enabling monitoring, early warning, and decision-making for fatigue risk control.

Despite this progress, implementation remains challenging because organizations need tools that can represent fatigue as a time-dependent process and translate complex determinants into actionable controls such as roster design, work-rest scheduling, task allocation, and early warning triggers [5, 7]. Evidence from healthcare also indicates that fatigue risk management strategies face practical barriers and require implementation-focused designs, strengthening the argument for models that connect measurement to decisions [8].

These needs have accelerated research on dynamic modelling approaches for occupational fatigue. Recent work has developed worker-focused fatigue modelling for industrial environments and discusses modelling assumptions in relation to operational constraints [9]. At the same time, recent systematic evidence syntheses show expanding interest in measuring and evaluating fatigue and fitness for work, indicating that field-ready monitoring has become a central research direction and a prerequisite for deploying dynamic prediction tools at scale [10].

This need has driven rapid growth in dynamic modelling approaches capable of capturing temporal change, uncertainty, and feedback processes. Recent studies illustrate how dynamic fatigue modelling is being operationalized across heterogeneous work settings, including construction work fatigue prediction and monitoring [11–13]. long-haul aviation fatigue risk estimation [14] and fatigue recognition under extreme mining conditions [15]. Complementary modelling families have also been applied to time-dependent fatigue mechanisms and related outcomes in diverse worker populations, such as dynamic neurophysiological modelling of mental fatigue [16] and symptom-network approaches in firefighters [17].

However, significant gaps remain before dynamic fatigue models can be widely adopted as deployment-ready risk management tools. Common limitations include restricted occupational samples, limited longitudinal field validation, and inconsistent benchmarking when subjective and objective indicators are not validated together for the target work context [12, 15]. Recent reviews and empirical studies also emphasize that fatigue in workers is shaped by interacting determinants that extend beyond physiology and sleep alone, including organizational factors and contextual conditions, which many modelling pipelines still integrate only partially [3, 5].

Accordingly, to consolidate this rapidly growing and methodologically diverse evidence base and to strengthen its contribution to occupational fatigue risk management, this review adopts a structured synthesis that links modelling choices to implementation needs across sectors. We characterize the landscape of dynamic occupational fatigue modelling and then evaluate how modelling approaches differ in assumptions, data requirements, interpretability, and decision-support outputs that enable monitoring, early warning, and control selection. We then identify the most persistent limitations in the current literature and propose priorities for future development, emphasizing hybrid and implementable models that integrate physiological, psychosocial, and contextual determinants of fatigue [5, 7].

### Research questions

To advance occupational fatigue risk management, this review synthesizes evidence on how fatigue is modeled as a dynamic phenomenon across work sectors. The following research questions guide study selection, data extraction, and the synthesis of results:

1. What dynamic modelling approaches are used for occupational fatigue prediction and management across sectors?
2. How do these approaches differ in theoretical assumptions, data requirements, interpretability, and practical utility for fatigue risk management?
3. What gaps remain for hybrid models integrating physiological, psychosocial, and contextual factors to improve real-world implementation?

## Methods

### Inclusion and exclusion criteria

The study includes peer-reviewed articles published between 2019 and 2025, focusing on dynamic or time-dependent modeling approaches for occupational work fatigue prediction, monitoring, or management. Studies that do not model fatigue dynamics over time, focus on non-worker clinical populations, or fall outside the scope of occupational work fatigue were excluded.

### Data sources and search strategy

Records were first identified from Scopus (DOI-indexed) and managed using Watase Uake Tools [18] to support screening and PRISMA 2020 reporting. Complementary searches were subsequently conducted in PubMed, ProQuest, Wiley Online Library, ScienceDirect, and Google Scholar to enhance coverage. The search strategy combined keywords related to occupational/work fatigue and dynamic modelling, including: “system dynamics fatigue”, “predictive fatigue model”, “bayesian network fatigue”, “dynamic causal modeling fatigue”, “worker fatigue prediction”, and “predicting worker fatigue”. All references were managed in Zotero, and study screening and data extraction followed the PRISMA 2020 guidelines. To ensure scope consistency with occupational/worker fatigue and to strengthen retrieval of eligible studies, a supplementary search was performed through targeted searching and backward/forward citation tracking. This supplementary process also enabled the identification of eligible replacement studies when full-text assessment indicated that certain records addressed non-occupational fatigue constructs.

### Selection process

The study identification, screening, and reporting workflow was supported by Watase Uake Tools, a collaborative research software platform developed to support systematic literature analysis, article mining, and evidence synthesis [18], including built-in PRISMA 2020 flow diagram generation features [19]. The database search yielded 180 records. Prior to screening, 67 records were removed, including 1 duplicate, 58 records flagged as ineligible by automated screening (restricted to the 2019–2025 window), 6 records removed for other reasons represent studies excluded through Tier Q1–Q4 relevance filtering, meaning that only scopus-indexed (Q1–Q4) publications were retained, while non-indexed articles were excluded, and 2 records without abstracts. Consequently, 113 records were screened by title and abstract, and 97 records were excluded because they did not model fatigue dynamics over time, focused on non-worker clinical populations, or otherwise fell outside the scope of occupational work fatigue. Full texts were sought for 16 reports, of which 8 reports could not be retrieved due to access limitations (full articles unavailable). The remaining 8 reports were assessed for eligibility and all were included.

**Fig. 1:**
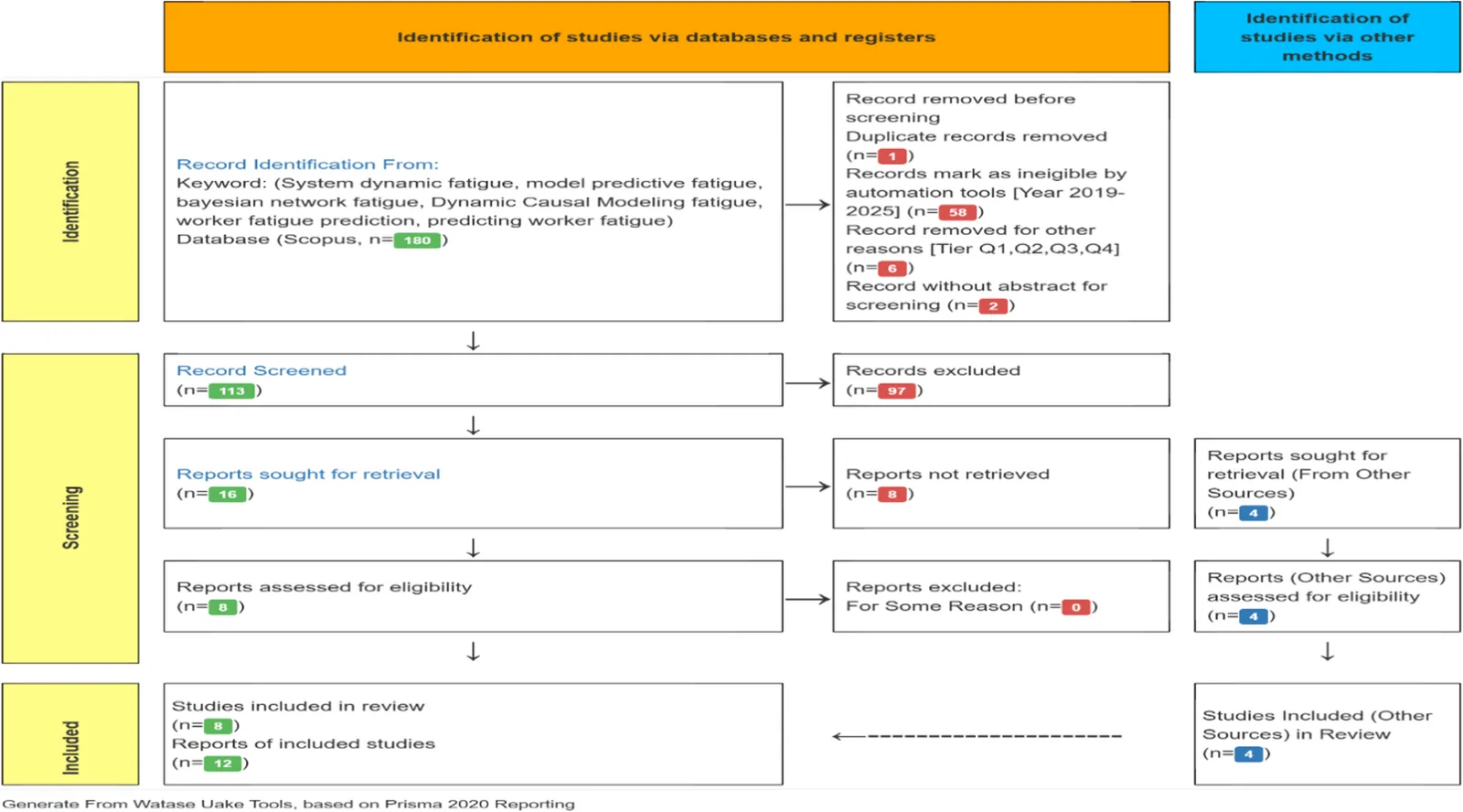
PRISMA flowchart of the literature selection process based on PRISMA 2020 guidelines

To ensure comprehensive coverage and replace records that were out of scope at full-text assessment, a supplementary manual search was conducted across Scopus, PubMed, ProQuest, Wiley Online Library, and ScienceDirect, resulting in 4 additional reports (“other sources”) that met the inclusion criteria. Altogether, the review included 12 articles (8 from database identification and 4 from supplementary manual searching), which formed the final evidence base for synthesis. Additional eligible studies were identified through manual searching in other databases using the same keyword strategy. In total, 12 articles were included in the final review, ensuring a comprehensive and systematic representation of dynamic approaches to modelling work fatigue. The study selection process is summarized in the PRISMA 2020 flow diagram (Fig. 1).

Following the study selection and data extraction procedures, the included studies were synthesized using a structured narrative approach aligned with the review objectives. To ensure coherence with risk management advancement, results are reported according to the three research questions: (i) mapping dominant dynamic modelling approaches across sectors, (ii) comparing approaches by assumptions, data needs, interpretability, and practical utility, and (iii) identifying gaps and future directions for more integrative and implementable models.

## Result

This systematic review synthesized evidence from twelve studies on dynamic approaches to model, predict, and manage occupational fatigue across sectors. The results are summarized in Tables 1, 2 and 3, covering study overview, methodological characteristics, and key findings with implications for fatigue risk management.

**Table 1:** Summary of protocol deviations

Author / Year	Country	Research Objectives	Research Context	Research Limitations	Grand Theory Used
Chen, Shoukun et al., 2025	China	To establish a dynamic fatigue recognition model for remote operators.	High-altitude, cold, low-oxygen mining environments	Small sample size, insufficient influencing factors, limited reference data for model validation.	Dynamic Bayesian Network, Hidden Markov Model
Bangaru, Srikanth Sagar et al., 2025	USA	To predict oxygen uptake during construction activities.	Construction activities involving manual labor.	Short monitoring time, only male participants, indoor setting.	Physiological Workload Theory, Aerobic Fatigue Threshold
Gao, B. et al., 2025	China	To assess driver fatigue using an interpretable dynamic recurrent framework from EEG features.	Driver fatigue assessment (EEG-based).	Generalizability across drivers and real-world conditions; dataset size constraints.	Time-series deep learning / dynamical systems
Zhou, Y. et al., 2025	China	To predict pilot fatigue risk during long-haul operations using Dynamic Bayesian Networks (DBN).	Aviation (long-haul flight fatigue risk).	Reliance on expert elicitation for priors; limited external validation.	Bayesian risk modelling (DBN)
Klyve, K. K. et al., 2023	Not specified	To incorporate a validated sleep/fatigue model with biological variability into nurse rostering to reduce highly fatiguing schedules.	Healthcare (nurse rostering / shiftwork).	Fatigue model approximation (look-up table) and computational scope may limit transferability.	Biomathematical sleep-based fatigue modelling
Umer, Waleed et al., 2023	UK, Hong Kong, Saudi Arabia	To assess the validity of the Borg-20 scale for fatigue monitoring among construction workers.	Construction industry, worker safety	Small sample size (*N* = 14); reliance on self-reported fatigue; potential environmental controls.	Physiological Monitoring
Pang et al., 2022	China	To establish a reliable and comprehensive prediction method for human fatigue in artificial atmospheric environments.	Artificial atmospheric environments, human fatigue	Limited sample size; focus on a controlled environment; potential variability in input parameters.	Dynamic Bayesian Network
Jebelli et al., 2020	USA, South Korea	To model and validate dynamic muscle fatigue generation and recovery for construction workers through laboratory testing.	Construction industry, upper limb fatigue	Focused on laboratory settings; small sample size; primarily male participants; potential variability in tasks.	Physiology and System Dynamics
Hosseini et al., 2019	Iran	To explore the effects of mental fatigue on brain areas through DCM parameters from EEG/ERP signals.	Mental fatigue, brain connectivity	Limited participant diversity; focus on EEG data without considering other neuroimaging techniques.	Dynamic Causal Modelling, EEG Analysis
Senouci, A. et al., 2022	USA	To develop a predictive tool for construction worker fatigue based on physiological and work-related indicators.	Construction worker fatigue prediction.	Single-context dataset and limited longitudinal tracking; need broader external validation.	Supervised predictive modelling (ANN / regression)
Liu, Y. et al., 2025	China	To characterize the fatigue–sleep cascade and related symptoms among firefighters using symptom network analysis and Bayesian DAG modelling.	Island-based firefighters (high-risk occupational group).	Cross-sectional design limits causal inference; generalizability beyond context requires study.	Network science / Bayesian DAG
Zong, H. et al., 2025	China	To develop an interpretable machine learning model to estimate fatigue levels of construction workers.	Construction worker fatigue modelling.	Need for multi-site validation and integration with time-dependent exposure histories.	Interpretable machine learning

**Table 2:** Methodology insights

Author / Year	Research Design	Research Focus	Data Collection Method	Research Methode	Dominant Analysis Technique
Chen, Shoukun et al., 2025	Quantitative	Study of fatigue characteristics of operators under extreme mining conditions.	Long-term field measurements of physiological signals.	Mixed methods analysis.	Dynamic Bayesian Network analysis
Bangaru, Srikanth Sagar et al., 2025	Quantitative	Predicting oxygen uptake in construction tasks.	Forearm IMU and EMG data collected from wearable sensors.	Machine Learning	BiLSTM Model Analysis
Gao, B. et al., 2025	EEG signal collection during fatigue assessment tasks.	EEG-derived features; fatigue state labels.	Interpretable Dynamic System Recurrent Network (IDSRN).	Model evaluation on labeled EEG dataset (accuracy/AUC).	Integrates interpretability with dynamic recurrent modelling for fatigue assessment.
Zhou, Y. et al., 2025	Expert evaluation and case study inputs for fatigue risk factors.	20 fatigue risk factors across human, machine, environment, and task.	Dynamic Bayesian Network with Noisy-OR conditional structure.	Case-based validation via posterior probability estimation.	Structured fatigue risk index system and DBN-based prediction under uncertainty.
Klyve, K. K. et al., 2023	Roster datasets validated sleep model outputs.	Shift patterns, sleep/wake dynamics, individual biological parameters.	MIP/CP with Large Neighbourhood Search; sleep model approximated via look-up table.	Computational experiments on real nurse rostering instances.	Operationalizes sleep-based fatigue dynamics for schedule optimization and personalization.
Umer, Waleed et al., 2023	Experimental Study	Validating methodological issues and improving fatigue monitoring tools.	On-site physiological monitoring	Longitudinal Study	Correlation and Kruskal-Wallis Tests
Pang et al., 2022	Experimental Study	Assessing human fatigue prediction accuracy and factors influencing fatigue.	Controlled simulation study with physiological measures.	Longitudinal Study	Bayesian Model and Analysis
Jebelli et al., 2020	Experimental Study	Understanding muscle fatigue accumulation and recovery during construction tasks.	Laboratory experiments on muscle fatigue	Longitudinal Study	System Dynamics Modelling
Hosseini et al., 2019	Experimental Study	Understanding effective brain connectivity during mental fatigue.	Continuous Performance Task	Longitudinal Study	Dynamic Causal Modelling
Senouci, A. et al., 2022	Physiological and survey/work-related data collection.	HR, sleep quality, workload-related indicators, individual factors.	Regression and ANN-based fatigue prediction tool.	Model validation using prediction performance metrics.	Provides a practical prediction tool linking physiological inputs to fatigue estimates.
Liu, Y. et al., 2025	Cross-sectional survey of firefighters.	PSQI, FSS, SCL-90, resilience indicators; symptom-level data.	Symptom network analysis and Bayesian Directed Acyclic Graph (DAG).	Network stability/centrality checks; exploratory DAG inference.	Identifies central symptoms and directional relations to guide targeted interventions.
Zong, H. et al., 2025	Data-driven modelling using construction worker indicators (field/lab dataset).	Workload-related measures and fatigue outcomes; key predictors identified via interpretability.	Interpretable machine learning approach (SHAP/ feature attribution).	Cross-validation and out-of-sample performance assessment.	Balances predictive accuracy with interpretability to support deployment in practice.

**Table 3:** Findings and implications

Author / Year	Research Novelty	Findings	Future Research Recommendations
Chen, Shoukun et al., 2025	Application of dynamic Bayesian networks for fatigue evaluation in miners in extreme environments.	The study demonstrated a reliable dynamic fatigue recognition model based on physiological signals and contextual information, with strong correlation to subjective fatigue reports.	Increase sample size, conduct multi-index tracking tests in future studies.
Bangaru, Srikanth Sagar et al., 2025	Development of a VO2 prediction model using wearable sensors.	The proposed BiLSTM model correlated strongly with measured VO2 and provided accurate predictions for oxygen uptake during scaffold building activities.	Implement the model in real construction sites, extend for different activities.
Gao, B. et al., 2025	Dynamic recurrent modelling of EEG can classify driver fatigue while providing interpretable system-level cues.	Supports real-time fatigue assessment and decision support for transportation safety.	Need validation across broader populations and real driving environments.
Zhou, Y. et al., 2025	DBN-based framework can estimate posterior fatigue risk under combined human machine environment task factors in long-haul flights.	Enables structured fatigue risk assessment for aviation FRMS and scenario-based prevention planning.	Requires larger empirical datasets and operational validation beyond expert-based priors.
Klyve, K. K. et al., 2023	Integrating a validated sleep model into rostering can reduce extreme fatigue exposure and personalize schedules.	Practical approach for designing safer shift rosters and improving alertness in healthcare staff.	Future work should test additional settings, refine approximations, and evaluate implementation feasibility.
Umer, Waleed et al., 2023	An on-site validation study revealing limitations in monitoring physical fatigue using physiological signals and the Borg scale in real world construction setting.	Correlation between physiological measures and fatigue is not as strong in real-world conditions; Borg-20 scale is not a suitable measure for fatigue; emphasizes need for multimodal monitoring for better accuracy.	Reassess physiological measures and fatigue models.
Pang et al., 2022	Development of a fatigue prediction method using a Dynamic Bayesian Network based on multiple factors.	The method provides a reliable means of predicting human fatigue, demonstrating that traditional measures may not suffice; it highlights the need for comprehensive multi-faceted approaches.	Improve prediction accuracy and expand the input features.
Jebelli et al., 2020	Development of a physiology-based dynamic fatigue model for upper limbs during construction tasks.	The model effectively predicts muscle fatigue changes in varying workloads, emphasizing the importance of addressing fatigue in construction settings for improved safety and health.	Investigate applicability to other muscles and task types.
Hosseini et al., 2019	Investigation of brain connectivity related to mental fatigue using dynamic causal modelling (DCM) of EEG data.	The study shows that mental fatigue influences effective connectivity among brain areas, revealing a complex interplay that can help design interventions for fatigue management.	Expand to include diverse populations and environments.
Senouci, A. et al., 2022	Physiological and work-related indicators can predict construction worker fatigue with useful accuracy.	Can inform proactive fatigue screening and tailored interventions on construction sites.	Needs longitudinal validation and integration with dynamic exposure histories and recovery.
Liu, Y. et al., 2025	Depression and daytime dysfunction centralize the fatigue–sleep cascade; DAG suggests directional relations among symptoms in firefighters.	Targets for interventions include sleep perception and resilience to mitigate fatigue-related dysfunction in high-risk workers.	Longitudinal studies are needed to confirm causal pathways and intervention effects.
Zong, H. et al., 2025	Interpretable ML can estimate fatigue levels and reveal key contributing factors for construction workers.	Supports transparent risk communication and actionable controls (work-rest, task design) in construction safety management.	Further research should incorporate time-dependent fatigue accumulation and multi-site deployment studies.

Across the included literature, four recurring modelling families remain prominent: (1) physiology informed dynamic simulation (including System Dynamics and biomathematical fatigue recovery formulations) to represent fatigue accumulation and recovery [20]; (2) data driven machine learning and deep learning models that infer fatigue-related states from physiological or contextual data streams [12, 21, 22]; (3) probabilistic dynamic models especially Dynamic Bayesian Networks to represent uncertainty and causal structure in fatigue risk prediction [14, 15, 23]; and (4) symptom-network/Bayesian graph approaches to characterize interrelations among fatigue and related outcomes in high-risk worker groups [17].

Table 1 highlights that the strongest construction-relevant evidence comes from studies that model physical workload and fatigue dynamics and apply monitoring or prediction in real or near-real construction activities, including physiology-informed fatigue modelling and field-oriented sensing and estimation approaches [11–13, 19, 21]. Complementary insights are provided by evidence from other high-risk or safety-critical contexts, such as plateau deep mining, long-haul aviation operations, healthcare shiftwork and rostering, firefighting, and transportation, which extends the review’s implications beyond construction and supports cross-sector transfer of modelling concepts and implementation considerations [14, 15, 17, 20, 22].

Table 2 shows a clear methodological shift toward study designs and analytical pipelines that better capture the time-dependent nature of occupational fatigue. The reviewed evidence increasingly relies on longitudinal or time-series data streams, drawing on field-based physiological monitoring, wearable sensing including IMU and EMG, neurophysiological inputs such as EEG, and integrated features reflecting interactions among the worker, task, and environment. Across studies, the dominant modelling approaches converge into three main families: machine learning and deep learning for near real-time fatigue prediction [13], Dynamic Bayesian Networks for uncertainty-aware estimation of evolving fatigue risk [24], and sleep-model-informed rostering and optimization designed to support system-level interventions through scheduling decisions [21].

Table 3 highlights a consistent shift from reactive fatigue management toward predictive and preventive decision support enabled by dynamic modelling. Across the included studies, model utility and predictive performance tend to be stronger when approaches integrate multimodal inputs and translate predictions into decision-ready outputs that can inform operational controls and fatigue risk management actions. Nevertheless, the evidence base remains limited by frequent reliance on single-source data, incomplete validation strategies, and insufficient benchmarking against both subjective fatigue ratings and objective indicators, underscoring the need for larger and more diverse samples, rigorous field-based validation, and deployment-oriented hybrid models that integrate physiological, psychosocial, and contextual determinants of occupational fatigue.

### Comparative analysis of dynamic modelling approaches

Physiology-informed dynamic simulation models are grounded in mechanistic representations of fatigue accumulation and recovery, typically requiring structured inputs such as workload, task duration, and sleep parameters. These models offer high interpretability and are particularly useful for scenario-based planning and scheduling decisions, but are limited in capturing real-time variability.

Machine learning and deep learning approaches rely on large-scale physiological or contextual data streams, including wearable sensor data, EEG signals, and environmental inputs. These models demonstrate strong predictive performance and enable near real-time fatigue monitoring. However, they often require extensive datasets and may suffer from limited interpretability, which can constrain their direct use in operational decision-making.

Probabilistic dynamic models, such as Dynamic Bayesian Networks, provide a balance between interpretability and predictive capability by modelling uncertainty and causal relationships. These models are particularly valuable for fatigue risk management systems, as they can integrate heterogeneous data sources and produce probabilistic risk estimates that support decision-making under uncertainty.

Symptom-network and Bayesian graph approaches focus on the interrelationships among fatigue-related symptoms and psychosocial variables. While these models enhance understanding of underlying fatigue mechanisms and target intervention points, their application in real-time fatigue monitoring and operational decision-making remains limited.

Overall, no single modelling approach fully addresses all requirements of fatigue risk management. Instead, hybrid models that integrate mechanistic understanding, real-time data processing, and probabilistic reasoning offer the greatest potential for implementation in complex occupational settings.

## Discussion

Across sectors, the updated evidence base demonstrates meaningful cross-industry relevance that can directly inform Fatigue Risk Management System (FRMS) design. Several modelling approaches developed in safety-critical settings translate well to occupational fatigue management in other industries because they address time-varying risk, incomplete information, and operational decision needs. Dynamic Bayesian Networks have been used to estimate and update fatigue-related risk in long-haul aviation operations, supporting uncertainty-aware decision-making under changing conditions [14]. Sleep-based fatigue modelling has also been integrated into nurse rostering to reduce highly fatiguing schedules, demonstrating how modelling can be operationalized as a scheduling control rather than only a monitoring tool [20]. Together, these studies underscore that fatigue management benefits from models that not only predict fatigue states but also produce outputs that can be embedded into safety controls and work planning.

**Fig. 2:**
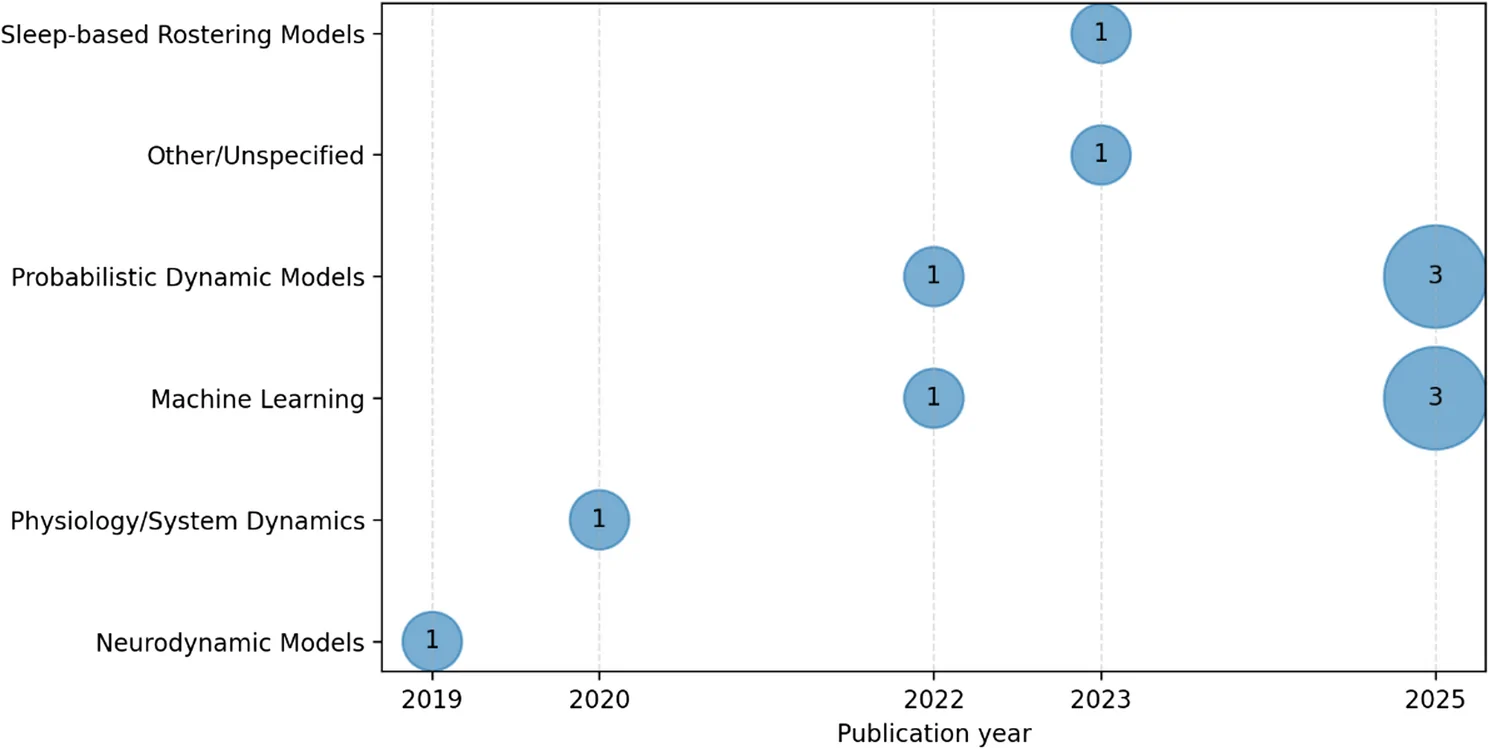
Trends in dynamic occupational fatigue modelling approaches (2019–2025)

Importantly, the distribution of modelling approaches over time indicates a shift toward data-driven prediction and probabilistic risk estimation, particularly in the most recent publications. This pattern suggests that contemporary fatigue management research is increasingly prioritizing operational readiness, including near real-time prediction capability and uncertainty-aware updating. At the same time, the smaller representation of mechanistic and scheduling-focused models implies that interpretability and scenario-based planning tools are less frequently developed in parallel, despite their direct value for fatigue control selection. This imbalance strengthens the case for integrated fatigue management systems that connect prediction outputs to defensible and actionable workplace controls. Temporal trends in dynamic occupational fatigue modelling approaches are presented in Fig. 2.

Beyond sectoral transferability, the reviewed studies reinforce that occupational fatigue is multi-component, involving physical load, cognitive depletion, and psychosocial strain. Neurophysiology-oriented approaches, including EEG-based modelling of mental fatigue mechanisms, highlight that fatigue dynamics may arise from distinct pathways that cannot be adequately captured using a single measurement modality. This strengthens the case for integrated modelling and monitoring strategies that jointly consider workload exposure, recovery opportunity, task and environmental conditions, and worker state indicators.

Key implementation considerations emerging from the updated corpus include limited sample sizes, short monitoring windows, and persistent challenges translating controlled findings to highly variable field conditions, as reflected across Tables 1, 2 and 3. In applied workplace contexts, several studies emphasize that multimodal monitoring combining physiological signals and contextual variables improves practical decision support because it better reflects real-world fatigue determinants. Equally important is the selection and validation of subjective fatigue measures; subjective scales should not be treated as universal benchmarks unless their validity is demonstrated for the specific work context and population, because inconsistent benchmarking undermines both model training and evaluation.

Across the updated studies, four persistent gaps constrain deployment readiness for fatigue management: limited external and field validation with small or context-specific samples; insufficient integration of biopsychosocial determinants alongside physiological workload and sleep opportunity; interpretability and transparency constraints for deep learning models in frontline decision-making; and inconsistent benchmarking when subjective scales are used without evidence of validity in the target work context,

Practically, these findings support the development of hybrid, deployment-oriented fatigue prediction systems for occupational settings. A layered architecture can combine complementary strengths: a System Dynamics layer to test workload and schedule scenarios and explore control options; a machine learning layer, such as bidirectional long short-term memory models, to estimate physiological workload or fatigue-related features from wearable time series; and a probabilistic layer, such as Dynamic Bayesian Networks or Hidden Markov Models, to update fatigue risk probabilities under uncertainty and incomplete data while providing decision-ready risk outputs. Such integration aligns with FRMS needs because it links prediction to control selection, supports continuous updating, and improves interpretability through mechanistic context. Overall, the updated synthesis indicates strong potential for integrated, multimodal, and interpretable dynamic models to enable proactive fatigue management across high-risk and safety-critical industries.

## Conclusion

This systematic review synthesizes dynamic modelling approaches used to understand, predict, and manage occupational fatigue, with clear practical relevance for fatigue risk management across occupational sectors. The updated evidence presented in Tables 1, 2 and 3 shows the increasing use of time-series-capable models and multimodal data, including wearables, physiological signals, and psychosocial measures, to support proactive fatigue risk management.

The review highlights the complementary strengths of several modelling approaches. System Dynamics offers value for scenario-based planning and policy simulation, machine learning and deep learning enable near-real-time fatigue prediction from sensor-based and physiological data, while probabilistic dynamic models such as Dynamic Bayesian Networks and Hidden Markov Models provide explicit handling of uncertainty, missing data, and evolving fatigue states. Together, these approaches can support more adaptive work-rest scheduling, targeted interventions, early warning systems, and better prioritization of fatigue controls in complex and safety-critical work environments.

From an implementation standpoint, future research should prioritize longitudinal field validation, harmonized benchmarking using both objective indicators and validated subjective measures, and interpretable model outputs that can be operationalized by supervisors, safety practitioners, and Fatigue Risk Management System teams. In particular, the development of hybrid fatigue models that combine dynamic simulation, predictive analytics, physiological and psychosocial inputs, and uncertainty-aware reasoning should be emphasized. Such models need to be tested in real occupational settings to ensure that their predictions are not only technically accurate but also understandable, actionable, and feasible for decision-making in high-risk industries.

These findings emphasize the need for hybrid, interpretable models that are validated in real-world occupational settings and capable of supporting actionable fatigue risk management decisions. Overall, this review contributes a structured synthesis of dynamic fatigue modelling methods and demonstrates how such approaches can strengthen FRMS design across high-risk and safety-critical industries.

## Recommendations

Based on the findings of this systematic review, the following recommendations are proposed for advancing research, practice, and policy related to fatigue management across occupational settings:

1. Future studies should conduct comparative benchmarking studies that evaluate multiple dynamic modelling approaches using the same datasets or operational scenarios to enable clearer assessment of model performance and applicability.
2. Researchers should translate the theoretical advantages of each modelling approach into practical tools that are accessible and actionable for safety managers and supervisors, including scenario planning dashboards, fatigue risk scores, and monitoring systems where feasible.
3. Developing hybrid models that integrate physiological monitoring, system-level simulation, and probabilistic reasoning is recommended to enhance predictive accuracy and support adaptive fatigue risk management strategies in complex, real-world occupational settings.
4. To address current research gaps, future work should emphasize longitudinal field validation, greater inclusion of biopsychosocial variables, and harmonized benchmarking using objective indicators alongside validated subjective measures.

## Data Availability

All data analyzed during this review are fully available within the text particularly in Tables 1, 2 and 3, which summarize the extracted information across included studies as well as in Study-level risk-of-bias reporting. Also included are the accompanying Supplementary Figure 1 and 2 search strategy and PRISMA.

## Abbreviations

ANN: Artificial Neural Network
AUC: Area Under the Curve
CP: Constraint Programming
DAG: Directed Acyclic Graph
DBN: Dynamic Bayesian Network
DCM: Dynamic Causal Modelling
DOI: Digital Object Identifier
EEG: Electroencephalography
EMG: Electromyography
ERP: Event-Related Potential
FRMS: Fatigue Risk Management System
FSS: Fatigue Severity Scale
HMM: Hidden Markov Model
HR: Heart Rate
IATA: International Air Transport Association
ICAO: International Civil Aviation Organization
IDSRN: Interpretable Dynamic System Recurrent Network
ILO: International Labour Organization
IMU: Inertial Measurement Unit
MIP: Mixed-Integer Programming
ML: Machine Learning
PRISMA: Preferred Reporting Items for Systematic Reviews and Meta-Analyses
PSQI: Pittsburgh Sleep Quality Index
SCL-90: Symptom Checklist-90
SHAP: Shapley Additive Explanations
VO₂: Oxygen Uptake
WHO: World Health Organization

## References

[1] World Health Organization. Long working hours increasing deaths from heart disease and stroke: WHO, ILO. 2021. https://www.who.int/news/item/17-05-2021-long-working-hours-increasing-deaths-from-heart-disease-and-stroke-who-ilo.

[2] International Labour Organization. Long working hours can increase deaths from heart disease and stroke, say ILO and WHO. 2021. https://www.ilo.org/resource/news/long-working-hours-can-increase-deaths-heart-disease-and-stroke-say-ilo-and.

[3] Cunningham TR, Guerin RJ, Ferguson J, Cavallari J. Work-related Fatigue: A Hazard for Workers Experiencing Disproportionate Occupational Risks. Am J Ind Med. 2022;65(11):913–25. 10.1002/ajim.23325. 35088430 PMC9325913

[4] Ropponen A, Gluschkoff K, Ervasti J, Kivimäki M, Koskinen A, Krutova O, Peutere L, Virtanen M, Härmä M. Working hour patterns and risk of occupational accidents: An optimal matching analysis in a hospital employee cohort. Saf Sci. 2023;159:106004. 10.1016/j.ssci.2022.106004.

[5] Safe Work Australia. Rapid review on evidence of managing the risks associated with fatigue. 2024. https://www.safeworkaustralia.gov.au/sites/default/files/2024-07/rapid_review_on_evidence_of_managing_the_risks_associated_with_fatigue.pdf.

[6] International Civil Aviation Organization. Manual for the Oversight of Fatigue Management Approaches (Doc 9966, Second Edition, Version 2 [Revised]). 2020. https://www.icao.int/publications/doc-9966-includes-complete-set-fatigue-management-implementation-manuals.

[7] International Air Transport Association. Fatigue Risk Management Systems (FRMS): White Paper. 2025. https://www.iata.org/contentassets/5f976bb3ca2446f3a40e88b18dd61fbb/frms_white-paper_2025.pdf.

[8] Fox S, Dall’Ora C, Young M. Fatigue risk management in healthcare: A scoping literature review. Int J Nurs Stud. 2026;174:105282. 10.1016/j.ijnurstu.2025.105282. 41273891

[9] Allemang-Trivalle A, Donjat J, Bechu G, Coppin G, Chollet M, Klaproth OW, Mitschke A, Schirrmann A, Cao CGL. Modelling fatigue in manual and robot-assisted work for operator 5.0. IISE Trans Occup Ergon Hum Factors. 2024;12(1–2):135–47. 10.1080/24725838.2024.2321460. 38441578

[10] Heng PP, Yusoff M, H., Hod R. Individual evaluation of fatigue at work to enhance the safety performance in the construction industry: A systematic review. PLoS ONE. 2024;19(2):e0287892. 10.1371/journal.pone.0287892. 38324557 PMC10849240

[11] Senouci A, Garimella SA, Kim K, Eldin N. A Model for Predicting Construction Worker Fatigue. J Smart Build Constr Technol. 2022;4(2). 10.30564/jsbct.v4i2.4628.

[12] Umer W, Yu Y, Afari MFA, Anwer S, Jamal A. Towards Automated Physical Fatigue Monitoring and Prediction Among Construction Workers Using Physiological Signals: An On-site Study. Saf Sci. 2023;166:106242. 10.1016/j.ssci.2023.106242.

[13] Zong H, Yi W, Chan APC, Yang H, Wu P, Xiao B. Developing a Fatigue Model for Construction Workers: An Interpretable Machine Learning Approach. J Manag Eng. 2025;41(4):04025025. 10.1061/JMENEA.MEENG-6662.

[14] Zhou Y, Chen D, Xiao J, Xiao Y, Lu Y, Zhang Y. A Pilot Fatigue Prediction Method Based on Dynamic Bayesian Networks. Hum Factors Ergon Manuf Serv Ind. 2025;35(3):e70011. 10.1002/hfm.70011.

[15] Chen S, Pan L, Xu K, Li X, Zuo Y, Zhou Z, Li B, Dai Z, Li Z. Real-time Monitoring and Prediction of Remote Operator Fatigue in Plateau Deep Mining Based on Dynamic Bayesian Networks. Sci Rep. 2025;15:1063. 10.1038/s41598-025-85316-4. 39774307 PMC11707074

[16] Hosseini GS, Nasrabadi AM. Technol Health Care. 2019;27(4):343–52. 10.3233/THC-181480. Effective Connectivity of Mental Fatigue: Dynamic Causal Modeling of EEG Data. 30932904

[17] Liu Y, Li Z, Xiang Q, Zhang X, Bai R, Sun C, Liu J. Depression and Daytime Dysfunction Centralize the Fatigue–Sleep Cascade in Island Firefighters: A Symptom Network and Bayesian DAG Study. Front Psychiatry. 2025;16:1663957. 10.3389/fpsyt.2025.1663957. 41234431 PMC12605024

[18] Wahyudi L. Watase Uake: Research Collaboration Tools. Retrieved January 29, 2026. 2024.from https://www.watase.web.id.

[19] Page, M. J., McKenzie, J. E., Bossuyt, P. M., Boutron, I., Hoffmann, T. C., Mulrow, C. D., … Moher, D. The PRISMA 2020 statement: An updated guideline for reporting systematic reviews. BMJ, 372, n71. 2021. 10.1136/bmj.n71. PMC8005924 33782057

[20] Jebelli H, Seo J, Hwang S, Lee S. Physiology-based Dynamic Muscle Fatigue Model for Upper Limbs During Construction Tasks. Int J Ind Ergon. 2020;78:102984. 10.1016/j.ergon.2020.102984.

[21] Klyve KK, Senthooran I, Wallace M. Nurse Rostering with Fatigue Modelling: Incorporating a Validated Sleep Model with Biological Variations in Nurse Rostering. Health Care Manag Sci. 2023;26(1):21–45. 10.1007/s10729-022-09613-4. 36197537 PMC10011308

[22] Bangaru SS, Wang C, Aghazadeh F, Muley S, Willoughby S. Fusion Sens. 2025;25(10):3204. 10.3390/s25103204. Oxygen Uptake Prediction for Timely Construction Worker Fatigue Monitoring Through Wearable Sensing Data. PMC12116003 40431996

[23] Gao B, Yan Y, Huangfu C, Cai J, Wang H. IDSRN: Interpretable Dynamic System Recurrent Network for Driver Fatigue Assessment. Appl Sci. 2025;15(21):11384. 10.3390/app152111384.

[24] Pang Z, Zhou Y, Zhao Y, Yang S, Pan Y. Method of Human Fatigue Evaluation in Artificial Atmospheric Environment Based on Dynamic Bayesian Network. Mathematics. 2022;10(21):3975. 10.3390/math10213975.

